# The Physician’s Visiting List, Diary, Etc., for 1860

**Published:** 1860-01

**Authors:** 


ART. IX.—*The Physician's Visiting List, Diary, and Book of Engagements, for* 1860. Philadelphia: Lindsay & Blakiston.

THIS book is much smaller than its New York competitor, and less full; but at the same time more portable, and, therefore, more convenient. It has, evidently, been prepared with much care, and will be found very useful to the practitioner in the daily rounds of his duties. It comprises, among other matters: An almanac; table of signs; Hall's ready method; poisons and their antidotes; table for calculating the period of utero-gestation; blank leaves for visiting lists; memoranda; addresses, for patients and nurses; obstetric engagements; vaccination engagements; and general memoranda.

